# From Cultural Tourism to Social Entrepreneurship: Role of Social Value Creation for Environmental Sustainability

**DOI:** 10.3389/fpsyg.2022.925768

**Published:** 2022-07-04

**Authors:** Xiaofeng Li, Jaffar Abbas, Wang Dongling, Noor Ul Ain Baig, Ruilian Zhang

**Affiliations:** ^1^School of Marxism, Hohai University, Nanjing, China; ^2^School of Media and Communication and Antai College of Economics and Management, Shanghai Jiao Tong University, Shanghai, China; ^3^Business School, Shandong Jianzhu University, Jinan, China; ^4^School of Management Sciences, Quaid-i-Azam University, Islamabad, Islamabad, Pakistan; ^5^Centre for Social Responsibility in Mining, Sustainable Minerals Institute (SMI), University of Queensland, Brisbane, QLD, Australia

**Keywords:** cultural tourism, social entrepreneurship, social value creation, environmental sustainability, cultural exchange

## Abstract

Cross-cultural exchanges among the locals and the tourists have been beneficial in terms of social value creation and sustainability. The present study has examined the role of cultural tourism and social entrepreneurship on social value creation and environmental sustainability. The study has drawn a sample through a non-probabilistic convenience sampling method for desired data collection, as investigators approached tourists visiting the tourism destinations. The study reports data received with the help of tourists visiting cultural heritage in the Gilgit-Baltistan region of Pakistan. The study has employed the PLS_SEM approach for analysis purposes to draw the results. The findings showed a significant relationship between cultural tourism, environmental sustainability, and social value creation that significantly predicts environmental sustainability. The results revealed a significant positive association between social entrepreneurship, social value creation, and environmental sustainability. Besides, results showed that social value creation mediates the relationship between cultural tourism and environmental sustainability and social entrepreneurship and environmental sustainability. The study’s findings climax the importance of cross-cultural interactions that enriches the cultural understanding and gives new perspectives to the existing cultural traditions. Pursuing environmental sustainability through social value creation requires an excellent combination of the administrative and political collaborative strategy that integrates cultural tourism and social entrepreneurship in tourist destination development and aims to attain improved tourist attractions. Besides, this research identifies a significant effect of cultural tourism on environmental sustainability. However, the relationship between tourism and environmental sustainability is bidirectional. It might provide direction for further study. The findings deliver valuable insight into global cultural tourism and social entrepreneurship activities that provide tourism destinations for community development. This investigation produces a systematic and holistic research framework to help explore the influence of cultural tourism and social value creation on the environmental sustainability at tourism destinations. The generalizability of the findings supplies helpful directions for future research on environmental sustainability related to social entrepreneurship and cultural tourism that leads to social value creation.

## Introduction

Scholars have debated culture and tourism. It shows the intertwined between tourism and culture for centuries (Li et al., 2021; Aman et al., 2022; Fu and Abbas, 2022; Ge et al., 2022; Liu et al., 2022). Cultural attractions, sights, and activities have always been essential motivations for travelers (Abbas et al., 2021; Wang et al., 2021; Mamirkulova et al., 2022). However, Richards (2018) argues that culture is the source of travel. Cultural tourism has become a broader subset of tourism worldwide, expanding its scope to include monuments and sites, lifestyles, creativity, traditions, and everyday culture (Noonan and Rizzo, 2017). Over the past decade, many broader themes have emerged, such as cultural and historical heritage, gastronomy, religion, and festivals (Hussain et al., 2017; Abbas et al., 2019a; Aman et al., 2019a; Mamirkulova et al., 2020). These attractions are more valued by tourists due to their local history, beliefs, traditions, and culture, as tourists recognize them for the cultural and religious mysteries attached to the tourists’ destinations (Lin et al., 2021). Likewise, cultural tourism has considerable potential to improve the sustainability of tourist attractions (Aman et al., 2019b). Tourism, especially cultural tourism, is a significant contributor to economic development (Abbas, 2020; Mubeen et al., 2021; Zhou et al., 2021; Farzadfar et al., 2022). However, lapses or deviations in tourism management decisions lead to a positive or negative influence on society and the environment (Buckley, 2012; Hallak et al., 2015; de Lange and Dodds, 2017; Kato, 2018; Parga Dans and Alonso González, 2019; Agarwal et al., 2020; Figueroa-Domecq et al., 2020). Recent studies have demonstrated a growing interest among researchers in cultural tourism. Studies identified gaps in the scientific literature on the role of social entrepreneurs in creating social value for tourists, which influences the development of environmental sustainability in tourism destinations (Heidari et al., 2018; Naderi et al., 2019; Parga Dans and Alonso González, 2019). Accordingly, this study investigates the significance of predicting the role of cultural tourism and social entrepreneurship in developing environmental sustainability (Cheng et al., 2021).

Consequently, cultural tourism influences the social value created by the social enterprises that help predict environmental sustainability (Hussain et al., 2019). Social entrepreneurship, a distinctive approach to solving economic and social problems, has recently attracted the attention of researchers due to its positive influence on communities, especially in developing countries (Abbas et al., 2020; Mubeen et al., 2020; Azadi et al., 2021; Azizi et al., 2021; Hussain et al., 2021). A past study explains the critical role of social entrepreneurship in the hospitality and tourism industry (Abbas et al., 2019b; Aqeel et al., 2021a; Maqsood et al., 2021; Rahmat et al., 2022). Social entrepreneurship and cultural tourism play a distinct and vivid role in attracting tourists to economic and cultural development destinations (Naderi et al., 2019). Scholars consider cultural tourism a vital pillar of the tourism industry. Cultural tourism refers to specific tourism that motivates tourists to explore and experience cultural attractions in the tourist destination places. Beliefs and culture are generally specific to a region. It has distinct attractions that lead to developing tourism behavior and the flow of tourists, generating income for residents (Kalvet et al., 2020). Social scientists have focused extensively on exploring cultural heritage, its conservation, local communities, tourism management, and documenting experiences and conflicts that cause damage and negative impacts on heritage sites (Parga Dans and Alonso González, 2019; Frey and Briviba, 2021). There is a pressing need to explore the relationship between the cultural and social perspectives of tourism that contribute to the sustainability of the surrounding environment. Although the tourism literature is vibrant; however, only a few local communities are aware of the importance of the social value created by social entrepreneurs. Nevertheless, its full potential has not been fully exploited in achieving a sustainable environment. Likewise, the importance of social entrepreneurship in tourism is even more critical due to its role in the sustainable development of the hospitality and tourism industry, as it has the potential to create social value for cultural tourists (Heidari et al., 2018). Besides, scholars emphasized measuring the environmental impacts of cultural tourism, and the current literature has highlighted its cultural and economic benefits for the local community (Spenceley, 2005).

Therefore, this study fills the identified gaps in cultural tourism and social entrepreneurship literature by proposing a comprehensive research framework for measuring the impact of cultural tourism and social entrepreneurship on the environmental sustainability of destination places. This descriptive research currently reports on cultural tourism in Pakistan. It proposes a conceptual model that provides a unique mechanism for achieving environmental sustainability through cultural tourism, and social entrepreneurship through consequent social value creation.

## Theoretical Background

When individuals travel, they often fundamentally learn about specific tourism locations rather than touring randomly. Research also shows that social tourists spend more cash and travel longer than typical tourists travel (Quan and Wang, 2004). Accordingly, understanding the inspiration of social tourists is crucial for cultural tourism scholars. Past literature has discovered that cultural tourists’ self-identity is often built through their cultural tourism motivations (Fistola and La Rocca, 2018; Ezenagu, 2020; Kalvet et al., 2020). The current study is based on self-determination theory (SDT), and past literature has broadly used it to explain the motivations of cultural tourists. According to this theory, individuals tend to seek new knowledge in their lives to help them learn and integrate their experiences or to seek inevitable consequences (Hsu, 2013; Lapan et al., 2016; Buzinde, 2020). This theory (SDT) describes that various factors motivate tourists that modulate their propensity to travel. For instance, inner pleasure, their urge to explore new destinations and participation in cultural tourism, engaging in cultural tourism for personal gain, and the expected monetary return of tourists associated with specific non-profit organizations may be possible motivations for tourists to engage in cultural tourism (Chen and Rahman, 2018).

Different degrees of motivational factors of individual tourists contribute differently to their cultural engagements with local communities. Other types of motivations may play different roles in the environmental sustainability of cultural tourism sites. A study by Ntoumanis (2005) described the positive results of SDT determining factors of personal motivation (Ntoumanis, 2005). For instance, the degree to which tourists’ needs are met, they will be more likely to be satisfied with the service provided and predict self-determined motivations such as intrinsic, extrinsic, and fixed (Chen and Rahman, 2018). Consequently, the self-determined motivation of the tourists would influence their intention to participate in future cultural tourism events (Ntoumanis, 2005). In this study, self-determination theory aims to motivate cultural tourists to engage in such behaviors when visiting cultural attractions that maintain the context of their destinations. At the same time, social value-creating social entrepreneurial behaviors will encourage tourists to revisit these destinations in the future.

### Cultural Tourism

Cultural tourism determines tourism activities where the primary motivation of tourists is learning, discovering, experiencing, and consuming tangible and intangible cultural products, services, and attractions of tourism destinations (Mamirkulova et al., 2020, 2022; Abbas et al., 2021). Such outcomes and attractions are related to a distinct set of spiritual, intellectual, and emotional characteristics of society, including architecture, literature, music, innovative industries, historical heritage, culinary heritage, cultural heritage, and living human culture. It includes its ways of spending life, values, beliefs, and traditions (Hussain et al., 2017; Aman et al., 2019b; Abbas et al., 2021; Aman et al., 2022; Farzadfar et al., 2022). Cultural tourism is a relatively new emerging concept in the field of the tourism industry (Richards, 2018). The literature on cultural tourism is very scattered. Although scholars have conducted studies on tourism; however, these studies reported on different directions and regions. Some studies have explored the economic impact of cultural tourism (Torre and Scarborough, 2017), and have systematic reviews (Richards, 2018). Scholars have investigated various aspects, such as the revival of the historic paths (Fistola and La Rocca, 2018), social capital (Martínez-Pérez et al., 2019), tourism experience, and destination loyalty (Chen and Rahman, 2018).

Some studies focused on exploring tourism impacts, such as achieving sustainability through tourism (Figueroa-Domecq et al., 2020), domestic cultural tourism (Castañeda et al., 2019), policy proposals to cope with excessive tourism (Frey and Briviba, 2021), tourism experiences (Seyfi et al., 2020), and cultural tourism as heritage resources (Ezenagu, 2020). The development of cultural tourism boosts the income growth of host countries, and the maintenance of tourist attractions helps stimulate the economy and preserve cultural heritage by attracting domestic and global tourists (Torre and Scarborough, 2017; Richards, 2018; Lin et al., 2021). When tourists visit different tourism destinations to explore the site’s attractions, their excessive traffic flow might damage cultural tourism sites (Parga Dans and Alonso González, 2019). Tourists’ travel motivations vary based on visitors’ interests, safety, wellbeing, and destination attractions. According to the fresh literature, the most visited tourist attractions are Portugal, Spain, France, and Italy due to their historical buildings, art, music, religion and cultural heritage, spiritual attribution, gastronomy, pilgrimages, cultural events, and festivals (Kalvet et al., 2020; Lin et al., 2021). Cultural tourism has gained widespread theoretical recognition from researchers as it plays a role in reconciling tensions between localities, ethnicities, and countries. It helps balance tourism management, heritage conservation, social pressures, and some aspects of economic development that represent a broader paradigm and appropriate approaches to environmental sustainability within tourist destination attractions (Buckley, 2012).

### Environmental Sustainability

Sustainability is considered an integral part of systems theory and is studied as an equation of balance between economic, environmental, and social aspects (Awan et al., 2019; Awan and Sroufe, 2019; Anser et al., 2020a; Ikram et al., 2021; Yang et al., 2021). It determines a fundamental premise of human activity (Begum et al., 2021a; Dhir et al., 2021). Sustainable development explains a complex evolving system that is subjectively adjusted to the specific circumstances and aspirations of the local community (Spenceley, 2005; Kato, 2018). The environmental sustainability of tourist attractions has essential implications for tourism management and cultural heritage protection (Begum et al., 2021b; Ikram et al., 2021; Awan and Sroufe, 2022). A past study found that excessive tourist visits flow at destination attractions can negatively impact the environment and ecological settings (Frey and Briviba, 2021). A study shows that diverse teams and leadership are more likely to develop environmental strategies that contribute to sustainable mechanisms (Figueroa-Domecq et al., 2020). This study also found that women were more likely to focus on environmental sustainability when leading tourism enterprises ((Anser et al., 2020b; Latif et al., 2020; Awan et al., 2022; Kumar et al., 2022; Yang et al., 2022; Zaman et al., 2022). However, men had shown their interest in being part of economic sustainability (Pitafi et al., 2018, 2019, 2020; Anser et al., 2020b; Latif et al., 2020; Rashid et al., 2020). Likewise, another study recognized that international tourists significantly influence the CO_2_ emissions in the geographic region, both in the short and long term (Akadiri et al., 2019). Another study in Iceland found that tourism has contributed to the deterioration of overall environmental sustainability (Saviolidis et al., 2021). By enriching cultural tourism resources, we can obtain a safe and hygienic environment that helps encourage tourists to revisit in the future (Kanwal et al., 2019, 2020; Khan et al., 2020; Shang et al., 2022). It helps to achieve the sustainable development of tourist attractions and destinations (Lin et al., 2021). Scholars consider cultural tourism a significant predictor of environmental sustainability. Based on the above literature, this study formulated the following hypotheses regarding the role of cultural tourism in environmental sustainability.

H1: There is a significant relationship between cultural tourism and environmental sustainability.

### Social Value Creation

The recent literature describes social value as increasing benefits and reducing costs, not just economic benefits, through a system that strives to address social needs and social problems that goes beyond financial benefits (Miller et al., 2008; Zahra et al., 2009). When an organization’s social purpose is clear and purposeful inherent in its business model, the organization is called a social enterprise. A study described that social entrepreneurial activities add social value along with economic value, but the social value created by these businesses differentiates social businesses from traditional enterprises (Naderi et al., 2019). The literature has also discussed that the sustainability of World Heritage sites depends on probing social science controversies and designing a workable framework that addresses tourism sustainability. However, an extensive study identified elements of social value as decisive aspects of cultural tourism heritage conservation (Parga Dans and Alonso González, 2019). Another study emphasized the need for such social policies, highlighting and stressing the search for entrepreneurial approaches to address the COVID-19 crisis and incorporate the concept of social value creation into tourism (Ratten, 2020). Naderi et al. (2019) conducted research to explore the effects of the business, economic value creation which is considered an essential component, while social value creation is the primary factor in sustainability (Naderi et al., 2019). Based on the above debate, this study leads to the formation of the following hypothesis:

H2: There is a significant relationship between cultural tourism and social value creation.

H5: Social value creation significantly influences environmental sustainability.

The study conducted by Naderi et al. (2019) explained the roles of social enterprises, and economic value creation and found it vital components; however, social value creation plays a critical and primary role in environmental sustainability (Naderi et al., 2019). Considering the need for cultural tourism demand, this business model needs to adhere to the environment, and a sustainable ecosystem to add social value to cultural tourists, thereby attracting more traffic to local destinations. It should be adequate for tourism management (Parga Dans and Alonso González, 2019). Therefore, creating social value through social entrepreneurship is an important concern that should be examined in the tourism and hospitality industry. Keeping in view the discussion above, this paper formulated the following hypothesis:

H6: Social value creation mediates the relationship between cultural tourism and environmental sustainability.

### Social Entrepreneurship

Social entrepreneurship is an approach by various groups, people, entrepreneurs, and start-ups, in which they design, create funding, and execute solutions to different cultural, social, and environmental concerns. Social entrepreneurs generally seek to advance the broad cultural, social, and environmental goals associated with the volunteer sector to attempt poverty alleviation, improve healthcare systems and contribute to community development (Zaman et al., 2016). Social entrepreneurs are individuals or enterprises that take business approaches to solve the social problems of society (Thompson, 2002). While contributing to economic growth at the macro level, spending by tourists on the local cultural economy also has broader external benefits (Torre and Scarborough, 2017). Tourism, in particular, has been established as a fertile opportunity for start-up spin-offs and social entrepreneurial activities due to the dominance and high success rates of small and medium-sized enterprises with reasonably low barriers to entry into the industry (Li, 2008; Figueroa-Domecq et al., 2020). Social entrepreneurs inject spending into the domestic economy, facilitating cultural tourism to increase income for the local community (Dhir et al., 2021). According to a past study, social entrepreneurship is a type of business that aims to solve social problems in a viable and sustainable way, with the financial benefits of sustainable growth (Naderi et al., 2019). From the hospitality and cultural tourism perspectives, scholars emphasize including and valuing social entrepreneurs, as they make an essential contribution to not only the cultural and social products but also help in the development of the tourism destinations (Nasrolahi Vosta and Reza Jalilvand, 2014). Therefore, incorporating social entrepreneurship into hospitality and cultural tourism will help maintain and develop the local destinations through environmental sustainability. Guri et al. (2021) argued that the combination of ecologically conservative views and cultural tourism would significantly contribute to tourist attractions of the tourism destinations (Guri et al., 2021). Social enterprises consider cultural tourism with an environmental focus, emphasizing improved usage patterns of cultural sites, and the achievement of environmental sustainability helps design and guide tourism activities, thereby helping to protect attractions and ensure local economic development. Based on the literature discussed above, this study formulates the following hypotheses:

H3: Social entrepreneurship positively influences social value creation.

H4: Social entrepreneurship positively and significantly affects environmental sustainability.

The social value created by social enterprises includes volunteer activities in the form of social services, community contributions, business cooperation, and tourism destination image promotion (Andersson and Larsson, 2016). Social value is a set of essential services or products that businesses provide and consider their social goals for the environment. It includes promoting and developing communities, supporting equity policies, and solving social problems by helping those in need by removing barriers to the social inclusion of specific groups. With reference to, the debate mentioned above, this paper proposed the following hypotheses:

H7: Social value creation mediates the relationship between social entrepreneurship and environmental sustainability.

Based on the gaps identified in the new literature review, the study developed the following framework. The basic mechanism of this study is based on social entrepreneurship theory (El Ebrashi, 2013). This integration will provide a deeper understanding of the role that social entrepreneurial behaviors play in stimulating social value creation in tourist destinations, thereby compelling tourists to visit places that are important to them.

### Research Framework

**FIGURE 1:**
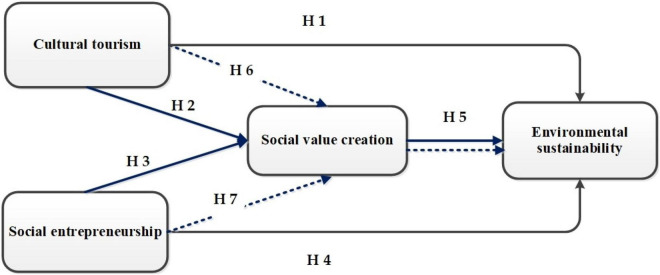
The formulated research model shows the variables of this study; cultural tourism, social entrepreneurship, social value creation, and environmental sustainability.

The present research study intends to explore the association between cultural tourism, social entrepreneurship, social value creation, and environmental sustainability. This survey explores how social value creation mediates the relationship between cultural tourism, social entrepreneurship, and environmental sustainability. The framework consists of four variables to show the proposed model. The study model selected cultural tourism and social entrepreneurship as independent variables (IVs), and environmental sustainability represents a dependent variable (DV). Concurrently, social value creation is the mediating variable. The present research attempts to address the identified literature gaps. The study evaluates the effects of cultural tourism, social entrepreneurship, and social value creation that develop tourists’ attitudes toward environmental sustainability. The following research model portrays a pictorial description demonstrating the theoretical grounds of this research study. Figure 1 below illustrates the proposed framework.

## Methodology

This study surveyed using a questionnaire to receive feedback from tourists over the age of eighteen years visiting Pakistan. The study included the respondents for data collection who had visited any historical or cultural tourism destinations and interacted with local social enterprises during their visits. The geographical area covered in this study is Gilgit-Baltistan and Azad Jammu and Kashmir region, as it has always been an internationally recognized tourist destination in Pakistan (Iqbal et al., 2019).

The researchers discussed the designed questionnaire with 15 students and two professors from tourism departments of different universities in Pakistan. The study performed a face validity analysis of the questionnaires. After discussions with students and tourism experts, the investigators included verbal suggestions in the questionnaire. The cover letter of this survey had a definition and brief facts about cultural tourism in accordance with the study’s operational purpose to avoid ambiguity. During the questionnaire feedback, the investigators requested the tourists to mention their recent journey in cultural tourism. Investigators distributed a total of 500 questionnaires to tourists and received back 338 valid questionnaires duly filled out. This study measured factor loadings, variable reliability, and validity, correlations, multicollinearity, and effect sizes modeled by structural equations. The study evaluated these tests using the Smart PLS 3.3.3., SmartPLS GmbH, Oststeinbek, Germany. The measured items indicated a decent outcome.

The self-administered questionnaires used for data collection included the chosen variables; cultural tourism, social entrepreneurship, social value creation, and environmental sustainability, which are in the growing scientific literature (Aqeel et al., 2021b,^2022^; Paulson et al., 2021; Mubeen et al., 2022; Yao et al., 2022). In contrast, the demographic variables included tourists’ ages, gender, education, income, ethnic/local, and occupational background. The study received the data by surveying tourists visiting Gilgit-Baltistan, Azad Jammu, and Kashmir. The study has drawn a sample through a non-probabilistic convenience sampling method, as investigators contacted tourists visiting these areas. The researchers asked visitors/tourists to fill out questionnaires if they had no objection and offered their consent for data collection. The investigators addressed respondents’/tourists concerns about anonymity by not revealing their identities.

### Measurement of the Instruments

The present survey adapted measurement scales from the existing literature. Scholars have broadly used it previously, and past studies extensively validated these selected instruments. However, this study re-examined instruments’ reliability and validity to ensure this study’s unbiased results.

### Cultural Tourism

This research used a scale for the first independent variable (cultural tourism) previously used in the recent literature to understand tourists’ interests and inclinations toward culturally and historically important tourism destinations. The study used an 11-item scale from the literature to measure the impact of cultural tourism (Chen and Rahman, 2018).

### Social Entrepreneurship

Similarly, this study used a measurement scale to assess the influence of social entrepreneurship on the second independent variable (social entrepreneurship). The survey has used 8-items from a past study (Naderi et al., 2019). The investigators modified statements of social entrepreneurship according to the context of cultural tourism. For instance, the item “Social enterprises try to increase their social impact and/or to serve their beneficiaries” (Chen and Rahman, 2018).

### Social Value Creation

The study evaluated the effects of the mediating variable (social value creation) by using a four-item scale previously used by Naderi et al. (2019) in the literature. The measurement items indicated a decent score (Naderi et al., 2019).

### Environmental Sustainability

This current survey measured the impact of the independent variable (environmental sustainability) by using a five-item scale adapted from the previous study by Biasutti and Frate (2017) broadly used in the literature (Biasutti and Frate, 2017).

## Results

Table 1 shows the demographics of the respondents/tourists. The location of tourists is precisely the leading tourist attraction destination in the Gilgit-Baltistan (GB) region of Pakistan.

**TABLE 1:** Demographic analysis of the tourists.

Demographics	Frequency	Percentage
**Gender**		
Male	192	56.97%
Female	145	43.02%
**Age (years)**		
18 – 30	118	35.01%
31 – 40	108	32.04%
41 – 50	67	19.88%
Above 50	44	13.05%
**Education**		
Bachelors	186	55.19%
Masters	101	29.97%
Ph.D. and others	50	14.83%
**Income**		
Less than $20,000	106	31.45%
$20,000 to $39,999	80	23.73%
$40,000 to $59,999	65	19.28%
$60,000 to $79,999	33	9.79%
$80,000 to $99,999	18	5.34%
More than $100,000	35	10.38%
**Origin of tourist**		
National	281	83.38%
International	56	16.61%
**No. of cultural destinations visited in last 5 years**		
1	98	29.08%
2 – 5	115	34.12%
6 – 10	75	22.25%
More than 10	49	14.54%
**Visiting companionship**		
With friends	89	26.40%
With family	102	30.26%
With touring organization	146	43.32%

*N = 338.*

The demographics of the tourists/participants revealed that most of them were local tourists residing within the country, while only 16% were international tourists. The lower flow of the global tourists was the result of the post-COVID-19 situation and fears of virus transmission as Pakistan is on the orange list. However, most tourists’ ages range between 18 and 30 years. The majority of tourists’ annual income was less than 20,000 US dollars. Since most of the visitors were up to 30 years, they belonged to early careers and did not have sufficient income. Table 1 provides the demographic profile of the recruited tourists.

### Measures, Items, Descriptive Stats, and Reliabilities

Table 2 shows the measurement items for the selected variables in the model, mean scores, standard deviation, skewness, and kurtosis results. This study selected a sample based on the ratio of the sample size to its free parameters (Golroudbary et al., 2019). The total parameter in the study was 28. According to the recommendation of an investigation by Bentler and Chou (1987), the ratio range is 5:1 to 10:1 (Bentler and Chou, 1987). Accordingly, the present study contains 28 free parameters, which indicates that a sample size between 140 and 280 appears sufficient. Thus, the sample size of this study is 338, which is enough to rule out any ambiguities for the sample size. It is in line with the recommendations of the recent literature. Table 2 shows the measurement items for the selected variables in the model, mean scores, standard deviation, skewness, and kurtosis results. The investigators screened and checked the received forms from the tourists. The researchers reviewed the data normality, skewness, and kurtosis outcomes. After screening, this study applied the smart PLS 3.3.3 for analysis purposes. The study results showed mesokurtic distribution and univariate normality. Many past studies showed that using the SEM approach produced a similar trend in their data sets, the usual outcome (Chen and Rahman, 2018). Table 2 shows measurement, items, and descriptive statistics.

**TABLE 2:** Measurement, items, and descriptive statistics.

Measurement	Items	Mean	SD	Skewness	Kurtosis
**Social entrepreneurship** (Naderi et al., 2019)	Social enterprises tries to increase their social impact and/or to serve their beneficiaries	4.003	1.023	−1.29	1.416
	This enterprise provides new ways to solve social problems come up very frequently	3.911	1.08	−1.084	0.709
	This enterprise places a strong focus on partnerships with other enterprises and/or governments in order to ensure a greater and accelerated accomplishment of the social mission	3.878	1.016	−0.93	0.449
	We are not afraid to take substantial risks when serving our social purpose	3.902	1.196	−1.034	0.081
	Organization tries to increase their social impact and/or to serve their beneficiaries	3.81	1.062	−0.87	0.15
	The enterprise provides new ways to solve social problems come up very frequently	3.864	1.116	−1.131	0.668
	The enterprise places a strong focus on partnerships with other enterprises and/or governments in order to ensure a greater and accelerated accomplishment of the social mission	3.772	1.129	−0.959	0.239
	The enterprises are not afraid to take substantial risks when serving their social purpose	3.769	1.183	−1.011	0.267
**Social value creation** (Naderi et al., 2019)	This enterprise continuously creates social benefits for its beneficiaries	3.620	1.058	−0.914	0.249
	This enterprise gives adequate contributions to the local community	3.715	1.128	−0.793	−0.164
	This enterprise continually improves the quality of its product and/or services	3.656	1.203	−0.934	0.006
	This enterprise plays a role in the local community that goes beyond the mere generation of profits	3.801	1.121	−0.934	0.214
**Environmental stability** (Biasutti and Frate, 2017)	When people interfere with the environment, they often produce disastrous consequences	3.798	1.117	−1.008	0.363
	Environmental protection and people’s quality of life are directly linked	3.772	1.142	−0.9	0.147
	Biodiversity should be protected at the expense of industrial agricultural production	3.721	1.073	−0.715	−0.114
	Building development is less important than environmental protection	3.852	1.248	−1.07	0.181
	Environmental protection is more important than industrial growth	3.653	1.13	−0.613	−0.466
**Cultural tourism** (Chen and Rahman, 2018)	I like to learn about different customs, rituals and ways of life	3.751	1.118	−0.751	−0.147
	I like to experience more than just staged events associated with this culture (e.g., dances)	3.688	1.068	−0.777	0.082
	I would like to get to know more about this culture	3.591	1.07	−0.632	−0.228
	I prefer just to observe how this culture is different rather than really meet and interact with people from that culture	3.644	1.142	−0.64	−0.433
	I am interested in getting to know more people from this culture	3.733	1.111	−0.774	−0.157
	The more I see, hear, and sense about this culture, the more I want to experience it	3.706	1.081	−0.781	−0.022
	I am very keen on finding out about this culture	3.783	1.049	−0.857	0.354
	I would like to see the world through the eyes of people from this culture	3.944	1.171	−1.094	0.374
	I like to spend time on finding out about this culture	3.688	1.04	−0.685	0.014
	I would like to get involved in cultural activities	3.653	1.098	−0.67	−0.171
	Contact with this culture forms a very important part of my experience in this visit	3.736	1.137	−0.71	−0.384

*N = 338.*

### Measurement Model

This present research study evaluates the merit and quality of the measurement model’s goodness to affirm variables’ reliability as well as the validity of analysis procedure output through the PLS-SEM technique. With reference to the previous argument, the study aims to measure the reliability, concurrent validity, and discriminant validity of each item of the chosen variables before testing and confirming proposed hypothetical statements (Hair et al., 2014, 2017, 2020; Sarstedt et al., 2017). A measurement model is part of a research framework that explores the connection between a study’s latent variables and their measurements. The SEM (structural equation modeling) approach explains a model, which quantifies the relationship between observations (indicators), acquired during a research survey, study, and hypothetical underlying structures or elements. This study evaluated the competence and efficiency of the proposed measurement model through confirmatory factor analysis (CFA). The refreshed literature has indicated a minimum threshold value of 0.4 for factor loadings (Bentler and Chou, 1987). However, all parameter values obtained in this study were above the threshold scores, with a minimum value of 0.681 for parameter SE5; the remaining values for all the items in this study met the acceptance criteria of a factor loading at 0.05. The model fit index shows an SRMR (Standardized Root Mean Square Residual for Structural Equation Modeling) is 0.08, which falls within the acceptable range based on past research (Cho et al., 2020).

In the next phase, the study examined the predictive relevance of the model (Q^2^), and the outcomes of the selected variables showed values above zero that confirmed the predictive relevance of the model (Benitez et al., 2020). The study results showed a decent value of Q^2^ for the dependent variables, environmental sustainability (*Q*^2^ = 0.465), and social value created (*Q*^2^ = 0.381). The results are significant and satisfactory. Likewise, the outcome of the model’s effect size (*f*2) indicated the overall significance of the model. It shows that the linear regression model designates a better fit to the data than a model (Benitez et al., 2020). Cultural tourism is an independent variable that shows a small effect on social value creation (*f2* = 0.024), while the dependent variable is environmental sustainability (*f2* = 0.053). However, social entrepreneurship has shown a large effect size on social value creation (*f2* = 0.389) and environmental sustainability (*f2* = 0.326). R2 shows variance measurement that is explained by the endogenous variables. Hence, R2 helps evaluate the study model’s predicted precision. The study results reveal *R*^2^ = 0.653 for social value creation, which shows a regression fit of 52.4%. Likewise, environmental sustainability shows a regression fit of 65.3%. The results are appropriate and show satisfactory outcomes. Hence, the study model looks adequate, sample size and all measured items show satisfactory results.

### Validity and Reliability of the Data

The study investigators screened and inspected the data received with excessive effectiveness. The study preliminary screened the feedback forms to perform further analysis for examining validity and reliability. The study measured the average variance extracted (AVE), Fornell and Larcker Criteria and HTMT (Heterotrait-Monotrait) ratio, and the variance inflation factor (VIF) and evaluated the discriminant and convergent validities. Besides, measured data reliability by performing Cronbach alpha and composite reliabilities. The reliability of the measurement scales obtained in this study fell within acceptable limits. The reliability threshold mentioned in the literature is 0.7 (Hallak et al., 2012). The current research shows that all items’ reliability values are higher than threshold limits.

The results indicated that social entrepreneurship revealed a value of 0.897, which means the internal consistency of the study variable, is adequate. According to the literature, a sufficient AVE value must be greater than 50%. The findings showed a satisfactory level of the model, as AVE outcomes are higher than the given threshold cut-off value of 0.5, which specifies a minimum value for the acceptance of the measurement scales (Dash and Paul, 2021). Similarly, the outer VIF statistics obtained from the measurement model fall under the cut-off value of 5 which provides adequate results (Craney and Surles, 2007). The values attained for the VIF do not show any severe issue of multicollinearity. Thus, all the items showed satisfactory outcomes. Table 3 shows item factor loadings, outer VIF, reliabilities, and AVE values.

**TABLE 3:** Items factor loadings, outer VIF, reliabilities, and AVE.

Item	CT	ES	Sent	SVC	Outer VIF
CT1	0.793				3.035
CT2	0.844				3.755
CT3	0.838				3.161
CT4	0.800				3.740
CT5	0.808				4.334
CT6	0.777				3.685
CT7	0.789				3.603
CT8	0.767				3.005
CT9	0.815				2.729
CT10	0.811				2.480
CT11	0.796				3.358
ES1		0.886			3.161
ES2		0.895			3.264
ES3		0.876			3.013
ES4		0.895			3.305
ES5		0.802			1.991
SE1			0.834		3.798
SE2			0.830		4.052
SE3			0.810		2.487
SE4			0.775		2.034
SE5			0.681		2.227
SE6			0.786		1.932
SE7			0.785		3.924
SE8			0.772		4.011
SVC1				0.827	2.154
SVC2				0.906	3.421
SVC3				0.908	3.390
SVC4				0.888	2.827
**Cronbach alpha reliability**	**0.945**	**0.920**	**0.897**	**0.905**	
**Composite reliability**	**0.953**	**0.940**	**0.917**	**0.934**	
**AVE**	**0.646**	**0.760**	**0.583**	**0.779**	

*N = 338; CT, cultural tourism; ES, environmental sustainability; SE, social entrepreneurship; SVC, social value creation. Bold values represent the satisfactory outcome.*

This study measured internal VIF values to eliminate multicollinearity problems. Thus, the study validated multicollinearity concerns between the chosen variables. Internal VIF values are satisfactory, which significantly explain the relationships between the study variables. It indicates that multicollinearity between the variables is irrelevant. Therefore, the model proposed in this study has sufficient convergent validity. Table 4 reports the values obtained by the internal VIF.

**TABLE 4:** Inner variance inflation factor (VIF) analysis.

	CT	ES	SEnt	SVC
CT		2.054	1.931	2.156
ES	2.729		2.184	2.801
SEnt	3.278	2.788		3.168
SVC	2.128	2.102	1.836	

*N = 338; CT, cultural tourism; ES, environmental sustainability; SEnt, social entrepreneurship; SVC, social value creation.*

The present study measured the discriminant validity of the data by the most widely used tests, such as the Fornell and Larcker Criteria and the HTMT ratios. The existence of discriminant validity indicates that the correlation between indicators of different variables is not well correlated that they lead the reader to conclude that they are measuring the same concept (Chen and Rahman, 2018). The Fornell and Larcker criteria show significant discriminant validity, as paired-wise squared correlations indicate that the highest paired-wise correlations are at the top of each column (Afthanorhan et al., 2021). Table 5 shows the study results regarding the Fornell and Larcker criteria.

**TABLE 5:** Fornell and Larcker criterion.

	CT	ES	SEnt	SVC
CT	**0.804**			
ES	0.666	**0.872**		
SEnt	0.708	0.791	**0.763**	
SVC	0.582	0.645	0.716	**0.883**

*N = 338; CT, cultural tourism; ES, environmental sustainability; SE, social entrepreneurship; SVC, social value creation. Bold values represent the satisfactory outcome.*

The results drawn through Heterotrait-Monotrait Ratio of Correlations (HTMT) indicated adequate levels and supported discriminant validity of the received data. The acceptability criteria based on the HTMT ratio determine that the generated values should be less than 0.85 (Franke and Sarstedt, 2019). Table 6 shows that all generated values are below the threshold limit of 0.85. Hence, it acknowledges discriminative validity and decency.

**TABLE 6:** Heterotrait-monotrait ratio of correlations (HTMT).

	CT	ES	SEnt	SVC
CT				
ES	0.706			
SEnt	0.742	0.846		
SVC	0.618	0.704	0.772	

*N = 338; CT, cultural tourism; ES, environmental sustainability; SE, social entrepreneurship; SVC, social value creation.*

### Path Coefficient and T-Statistics

Scholars broadly use the PLS-SEM tool to measure standardized beta (β) coefficient’s path coefficients (Hair et al., 2012b). Each unit variation, fluctuation, or change of the exogenous variables equals a likely variation/interpretation resulting from the endogenous variables. In the analysis results, path coefficients characterize these possible variations. Experts approximate and equate conceptual models based on the generated value of each path. Ultimately, the paths with greater values demonstrate considerably significant influence on endogenous variables of the model, and the lower values show weak or inadequate, or insufficient effects (Hair et al., 2014, 2020; Sarstedt et al., 2017).

The *t*-test help scholars in determining the comparison of means scores generated from two data sets that differ significantly from each other. Scholars widely incorporate the *t*-test approach to examine the path coefficient(s) significance level in the equation. Chin (1998) described that scholars use bootstrapping approach to determine the position or significance of the proposed hypothesis of the study (Chin, 1998). The statistical significance has been represented at the 5% level of significance, and the values of the t-statistics should be either equal to 1.96 or higher than the 1.96, that is the specific cutoff value to test hypothesis significance (Hair et al., 2012a). Table 7 here shows t-statistics to test the study’s proposed hypotheses.

**TABLE 7:** Direct effects and hypotheses testing.

Paths	H	O	M	SD	t-statistic	*p*-value	Results
CT → ES	H_1_	0.193	0.193	0.071	2.721	0.007**	***Accepted***
CT → SVC	H_2_	0.151	0.156	0.064	2.347	0.019*	***Accepted***
SE → SVC	H_3_	0.559	0.562	0.076	7.357	0.000***	***Accepted***
SE → ES	H_4_	0.610	0.607	0.056	10.921	0.000***	***Accepted***
SVC → ES	H_5_	0.132	0.128	0.062	2.114	0.035*	***Accepted***
CT → SVC → ES	H_6_	0.020	0.021	0.014	1.441	0.150	**Rejected**
SEnt → SVC → ES	H_7_	0.080	0.077	0.038	2.098	0.036*	***Accepted***

N = 338, ***p < 0.001, **p < 0.005, *p < 0.05, H, hypothesis; O, original sample; M, sample mean; SD, standard deviation; CT, cultural tourism; ES, environmental sustainability; SE, social entrepreneurship; SVC, social value creation. Bold values represent the satisfactory outcome.

### Hypotheses Testing

Hypothesis testing clarifies the study variables’ proposed relationships, showing how well variables correlate with each other. Bari et al. (2019) explained that the PLS-SEM approach (structural equation modeling) helps scholars to test the formulated hypothesis that clarifies the relationships between chosen variables of the study (Bari et al., 2019). Table 7 shows the theoretical paths hypothesized in this study to demonstrate the relationship between cultural tourism and social entrepreneurship with social value creation and environmental sustainability.

The first path indicates the proposed hypothesis, H_1_: There is a significant relationship between cultural tourism and environmental sustainability. The results as indicated in Table 7 validate this hypothesis (β = 19.3%, *t*-statistic = 2.721 and *p* < 0.05). The findings show a significant positive influence of cultural tourism on environmental sustainability. Results approved H_1_.

The second path shows the results of the formulated hypothesized statement (H_2_: There is a significant positive relationship between cultural tourism and social value creation). The results have approved and accepted this hypothesis. The study results have demonstrated a 15.6% change in social value creation with one unit change in cultural tourism. The results of the second hypothesis (β = 15.1%, *t*-statistic = 2.347 and *p* < 0.05) at 5% significance level validate H_2_.

Similarly, the third path shows the proposed hypothesis, H_3_: Social entrepreneurship positively and significantly influences social value creation. The study results confirm and validate the third hypothesis. The findings have demonstrated a 55.9% change in social value creation with one unit change in social entrepreneurship. The results of the third hypothesis show that social entrepreneurship has shown a significant impact on social value creation. The findings as shown in Table 7 (β = 55.9%, *t*-statistic = 7.357 and *p* < 0.001) validate and approve H_3_.

Likewise, the fourth path expresses the hypothesis, H_4_: Social entrepreneurship positively and significantly affects environmental sustainability. The results of the fourth hypothesis indicate that social entrepreneurship positively affects environmental sustainability. The findings as presented in Table 7 (β = 61%, *t*-statistic = 10.921 and *p* < 0.001) validate and accept H_4_.

This study formulated a fifth hypothesis, H_5_: Social value creation positively affects environmental sustainability. The study findings for the fifth hypothesis show that social value creation has a positive and significant influence on environmental sustainability, which shows that 13.2% change in environmental sustainability results from one unit change in social value creation. The findings as shown in Table 7 (β = 13.2%, *t*-statistic = 2.114 and *p* < 0.05) have validated H_5_.

Similarly, the study proposed the sixth hypothesis, H_6_: Social value creation mediates the relationship between cultural tourism and environmental sustainability. The sixth hypothesis results have not shown a strong mediating relation between cultural tourism and environmental sustainability. Similarly, cultural tourism has not shown a significant association with environmental sustainability. Based on the findings (β = 2%, *t*-statistic = 1.441) results have rejected H_6_.

Finally, this study proposed the seventh hypothesis, H_7_: Social value creation mediates the relationship between social entrepreneurship and environmental sustainability. The findings related to the seventh hypothesis have indicated a positive mediating connection between social entrepreneurship and environmental sustainability. Results have shown a significant mediating relationship between social entrepreneurship and environmental sustainability. Thus, based on Table 7 results (β = 8%, *t*-statistic = 2.098, and *p* < 0.05), the study validates H_7_. See Table 7.

## Discussion

This study explores the relationship between cultural tourism, social entrepreneurship, social value creation, and environmental sustainability. This research paper examines how social value creation mediates the relationship between cultural tourism, social entrepreneurship on environmental sustainability. Studies have shown that cultural tourism and social entrepreneurship have a significant and positive impact on social value creation, thereby significantly predicting environmental sustainability. In the context of tourism, more cultural tourism activities contribute to the literature on environmental sustainability and social value creation. The findings of this study are contrary to some previous studies that have shown environmental degradation in tourist attraction destinations (Frey and Briviba, 2021). Cultural tourists exhibit various motivation types to gather different experiences about cultural tourism destinations. The expected interaction with the local community is cited as one of the main motivations for tourists (Reisinger, 1994). This study suggests that the more tourists seek to understand a cultural perspective of a tourism destination, the higher their engagement with the destination’s environmental sustainability. A higher level of cultural tourism in the destination leads to better environmental sustainability, which is in line with the past literature. Several past studies have reported the same findings and emphasized exploring the relationship between cultural tourism in the destination and environmental sustainability (Pulido-Fernández et al., 2019). Tourists become more aware of protecting the environment and keeping it safe from excessive deterioration due to their presence or activities (Figueroa-Domecq et al., 2020). Therefore, tourist attractions seeking more cultural tourism should design local organizations that provide visitors with an experiential learning environment through education and engagement activities that help visitors maintain and moderate environmental sustainability.

Testing the formulated hypothesized statements elucidates the chosen variables’ links, showing how well study variables correlate with each other. The study shows the theoretical paths hypothesized to validate the association between cultural tourism and social entrepreneurship with social value creation and environmental sustainability.

The first path (H_1_) designates a significant association between cultural tourism and environmental sustainability. The results (β = 19.3%, *t*-statistic = 2.721 and *p* < 0.05) approved it.

The second path (H_2_) outcome revealed a significant positive connection between cultural tourism and social value creation. The study findings established a 15.6% change in social value creation with one unit change in cultural tourism. The results (β = 15.1%, *t*-statistic = 2.347 and *p* < 0.05) of the second hypothesis at 5% significance level approved H_2_.

Likewise, (H_3_) narrates that social entrepreneurship significantly influences social value creation. The findings verified a 55.9% change in social value creation with one unit change in social entrepreneurship. The results of H_3_ show that social entrepreneurship has shown a significant influence on social value creation. The results (β = 55.9%, *t*-statistic = 7.357 and *p* < 0.001) validate and approve H_3_.

Similarly, H_4_ stated that social entrepreneurship significantly affects environmental sustainability. The results of H_4_ (β = 61%, *t*-statistic = 10.921 and *p* < 0.001) indicated that social entrepreneurship positively affects environmental sustainability. The findings, as indicated in Table 7, validated and accepted H_4_.

This study’s H_5_ claims that social value creation positively affects environmental sustainability. The study findings show that social value creation has a positive and significant effect on environmental sustainability, which shows a 13.2% change in environmental sustainability results from one unit change in social value creation. Results (β = 13.2%, *t*-statistic = 2.114 and *p* < 0.05) have validated H_5_.

Similarly, H_6_ claims that social value creation mediates the relationship between cultural tourism and environmental sustainability. Thus, cultural tourism has not shown a significant relationship with environmental sustainability. The findings (β = 2%, *t*-statistic = 1.441) have rejected H_6_.

Finally, this study proposed H_7_. It describes that social value creation mediates the relationship between social entrepreneurship and environmental sustainability. The findings indicated a positive mediating linkage between social entrepreneurship and environmental sustainability. Results have shown a significant mediating relationship between social entrepreneurship and environmental sustainability. Based on the results (β = 8%, *t*-statistic = 2.098, and *p* < 0.05), the study validated H_7_.

The results further show that cultural tourism plays a vital role in creating the social value of tourist attractions. When cultural tourism enhances social capital in terms of networking and social connections with local communities, it automatically creates social value, generating more social benefits at the tourism destinations (Chen and Rahman, 2018). Cultural tourism activates tourism destination hosting institutions to voluntarily participate in social activities, including community contribution, social activities, and promoting the image of cultural destinations (Brandt et al., 2017; Martínez-Pérez et al., 2019; Parga Dans and Alonso González, 2019; Dahles et al., 2020; Kalvet et al., 2020). Therefore, a series of activities have been developed for cultural tourists to make their visit more experiential and memorable, which generates an urge to revisit the tourism destination spots. Thus, adding social value to the tourists motivates them for more tourism activities. Cultural tourists generally want to experience the beauty, novelty, uniqueness, authenticity, and beauty of a cultural destination. Tourists wish to gain easier access to knowledge about a tourism destination’s culture, atmosphere, and natural diversity of cultural places (Reisinger, 1994). Cultural tourism helps create experiences about food, local life, and cultural diversity, leaving unforgettable travel experiences in the minds of tourists (Chen and Rahman, 2018).

When social entrepreneurship is higher in tourist destinations, it creates enhanced social value between tourist destinations, which leads to elevated environmental sustainability. The current study provides a holistic understanding of social value creation through social entrepreneurship. The study argues that cultural tourism and social entrepreneurship offer a healthy environment that creates social value for tourists interested in learning and interacting with new cultures of tourism destinations. Past research has also found that social enterprises contribute to social value creation (Brandt et al., 2017; Dwivedi and Weerawardena, 2018; Hlady-Rispal and Servantie, 2018; Naderi et al., 2019; Sam Liu and Huang, 2020). However, the cultural tourism literature also recognizes the predictive role of social entrepreneurship in creating social value for cultural tourists. Besides, social entrepreneurship is a strong predictor of the environmental sustainability of cultural tourism sites. Previous research has also highlighted how enterprise management can have a significant impact on the environment at both the local and global levels (Spenceley, 2005).

Past research has used different environmental techniques to measure the impact and deterioration caused by various enterprises (Hallak et al., 2012; Wang et al., 2013; Bansal et al., 2019). Enterprises will need support and information to initiate, implement and maintain best environmental practices. When social enterprises have sufficient resources, goals, and object-oriented operations, they mechanically contribute to the social value creation and environmental sustainability of tourist attractions spots. Besides, the study found that there would be improved environmental sustainability in cultural tourism sites when there is a higher level of social value creation from social enterprises. When an enterprise integrates the corporate strategy of sustainable development into its business activities to achieve overall sustainable development, it reflects in the intangible assets of the organization in the form of social value (Ciasullo and Troisi, 2013). Social entrepreneurship implements sustainable corporate strategies, and value creation systems come into operation, addressing entrepreneurial development and environmental protection issues. Therefore, considering the problematic social issues that cultural tourism faces after COVID-19, it is crucial to make entrepreneurial decisions to create social value in society. Achieving a realistic ideal system in which the overarching goals of social value creation and entrepreneurship while making decisions that help maintain tourist destinations’ environmental sustainability and biodiversity is helpful for both enterprises and society.

### Practical Implications

Pursuing environmental sustainability by creating social value requires the perfect combination of administrative and political cooperation strategies. These factors integrate cultural tourism and social entrepreneurship in developing tourist destinations that help achieve the goals of improving tourist attractions. Cultural tourism is an absolute facilitator of geographic development if social value creation is adequately managed through community service and social entrepreneurship. Cultural tourism and social entrepreneurship are fundamental support for creating social value in the cultural tourism destination spots. Therefore, cultural tourism and social enterprises contribute a balance to society with environmentally sustainable. In addition, tourism destination stakeholders should recognize those cultural tourism factors that lead to increased tourism activities. Cultural tourism helps create social value and contributes to the environmental sustainability of tourist attractions. Thus, cross-cultural exchanges between the local community and tourists have always been beneficial in terms of social capital, financial inflows, or the enhancement of culture as a tourist attraction. This study suggests that improved government and administrative regulations and policies can leverage cultural tourism and social entrepreneurship to create value in tourist attractions, which will help preserve the destination’s biodiversity and natural resources.

### Limitations and Future Directions

This research study reports some limitations and directions like any empirical investigation. Although cultural tourism and social entrepreneurship have a significant and positive impact on social value creation, other factors may contribute to the mode of integration. Scholars can conduct studies to explore the implications of other factors in future research. Therefore, other latent variables such as memorable tourist experience, willingness to revisit, infrastructure development, etc., can be incorporated into the model to gain a deeper understanding of cultural tourism. In addition, this study found that cultural tourism has a positive and significant impact on environmental sustainability, but the relationship between tourism and environmental sustainability can be bidirectional. Future research studies can bring more exciting results with new models (Spenceley, 2005). This study only takes one province of Pakistan as the geographic location of the sample. Still, to expand the study’s geographic, cultural, and religious scope, other tourist destinations such as Kartarpur Corridor, Katas Raj Temple, and Nankana Sahib consider exploring the religious perspective of tourism. Besides, further surveys can also explore the results from Hassan Abdaal, another tourism destination in Pakistan. It could open up a new avenue for comparing results by region and religion. This current research is limited to the statistical analysis of results drawn from this sample. Since cultural tourism is an experience, the exposure to qualitative research will enhance the study’s understanding and scope by considering the study’s triangulation method, which will also provide a basis for validating the findings of this study.

## Conclusion

This study examines the impact of cultural tourism and social entrepreneurship on social value creation and environmental sustainability. The findings show that when cultural tourism and social entrepreneurship are higher, the tourism destination creates more social value. As a result, social value creation significantly and positively affects environmental sustainability. Tourism destination managers working in social enterprises can use tourists’ cultural tourism experiences to understand what tourists say about social and community services. The findings provide valuable insight into global cultural tourism and social entrepreneurship activities that provide tourism destinations for community development. This investigation produces a systematic and holistic research framework to help explore the influence of cultural tourism and social value creation on the environmental sustainability at tourism destinations. The generalizability of the findings supplies helpful directions for future research on environmental sustainability related to social entrepreneurship and cultural tourism that leads to social value creation (Abbas et al., 2019c). Literature shows that one of the goals of cultural tourism is to create a connection and interaction with tourists to create an overall social value that benefits the tourists and the local community. When social enterprises and cultural tourism authorities collaborate, it supports the creation of social value, helping the population at a broader level by maintaining natural environment resources. Therefore, governments and local authorities must facilitate interaction between hosts and tourists to enhance cultural exchanges and provide tourists with a pure experience.

## Data Availability Statement

The original contributions presented in this study are included in the article/supplementary material, further inquiries can be directed to the corresponding authors.

## Ethics Statement

Ethical review and approval was not required for the study on human participants in accordance with the local legislation and institutional requirements. Written informed consent from the patients/participants OR patients/participants legal guardian/next of kin was not required to participate in this study in accordance with the national legislation and the institutional requirements.

## Author Contributions

All authors listed have made a substantial, direct, and intellectual contribution to the work, and approved it for publication.

## Conflict of Interest

The authors declare that the research was conducted in the absence of any commercial or financial relationships that could be construed as a potential conflict of interest.

## Publisher’s Note

All claims expressed in this article are solely those of the authors and do not necessarily represent those of their affiliated organizations, or those of the publisher, the editors and the reviewers. Any product that may be evaluated in this article, or claim that may be made by its manufacturer, is not guaranteed or endorsed by the publisher.
